# The role of oxidant stress and gender in the erythrocyte arginine metabolism and ammonia management in patients with type 2 diabetes

**DOI:** 10.1371/journal.pone.0219481

**Published:** 2019-07-17

**Authors:** Martha L. Contreras-Zentella, Lourdes Sánchez-Sevilla, Juan A. Suárez-Cuenca, Marisela Olguín-Martínez, Martha G. Alatriste-Contreras, Norberto García-García, Lorena Orozco, Rolando Hernández-Muñoz

**Affiliations:** 1 Departamento de Biología Celular y Desarrollo, Instituto de Fisiología Celular; Universidad Nacional Autónoma de México (UNAM), Coyoacán, Mexico City, Mexico; 2 Departamento de Medicina Interna, Hospital General “Xoco”, Secretaría de Salubridad, Coyoacàn, Mexico City, Mexico; 3 Departamento de Métodos Cuantitativos, División de Estudios Profesionales, Facultad de Economía, Universidad Nacional Autónoma de México (UNAM), Coyoacán, Mexico City, Mexico; 4 Laboratorio de Enfermedades Inmunogénicas y Metabólicas, Instituto Nacional de Medicina Genómica (INMEGEN), Tlalpan, Mexico City, Mexico; East Tennessee State University, UNITED STATES

## Abstract

**Objectives:**

To study the differences in the levels of nitrogen metabolites, such as ammonia and nitric oxide and the correlations existing among them in both red blood cells (RBCs) and serum, as well as the possible differences by gender in healthy subjects and patients with type 2 Diabetes Mellitus (DM).

**Design and methods:**

This cross-sectional study included 80 patients diagnosed with type 2 DM (40 female and 40 male patients) and their corresponding controls paired by gender (40 female and 40 male). We separated serum and RBC and determined metabolites mainly through colorimetric and spectrophotometric assays. We evaluated changes in the levels of the main catabolic by-products of blood nitrogen metabolism, nitric oxide (NO), and malondialdehyde (MDA).

**Results:**

Healthy female and male controls showed a differential distribution of blood metabolites involved in NO metabolism and arginine metabolism for the ornithine and urea formation. Patients with DM had increased ammonia, citrulline, urea, uric acid, and ornithine, mainly in the RBCs, whereas the level of arginine was significantly lower in men with type 2 DM. These findings were associated with hyperglycemia, glycosylated hemoglobin (Hb A_1C_), and levels of RBC’s MDA. Furthermore, most of the DM-induced alterations in nitrogen-related metabolites appear to be associated with a difference in the RBC capacity for the release of these metabolites, thereby causing an abrogation of the gender-related differential management of nitrogen metabolites in healthy subjects.

**Conclusions:**

We found evidence of a putative role of RBC as an extra-hepatic mechanism for controlling serum levels of nitrogen-related metabolites, which differs according to gender in healthy subjects. Type 2 DM promotes higher ammonia, citrulline, and MDA blood levels, which culminate in a loss of the differential management of nitrogen-related metabolites seen in healthy women and men.

## Introduction

Diabetes mellitus (DM) is a worldwide disease frequently associated with a high risk of atherosclerosis and renal, nervous system, and ocular damage [1]. Reactive oxygen species (ROS) have been implicated in the pathogenesis of DM [2], as well as in its complications [3,4]. Patients with type 2 DM frequently have vascular endothelium dysfunction, which is related to hypercholesterolemia, and nitric oxide (NO) deficiency, a major factor that contributes to endothelial dysfunction [5].

Another source of possible complications in DM patients could be derived from an altered nitrogen metabolism, even in the absence of evident liver disease and/or nephropathy. Certainly, experimentally induced diabetic rats have lower gut amino acids utilization, which can diminish alanine and ammonia release into the portal circulation [6]. Therefore, this type of experiments could provide a background for the understanding of why DM patients with hepatic encephalopathy have a lower liver glucose absorption and utilization [7]. Indeed, it has been reported that DM patients have severe hepatic encephalopathy at early stages of chronic liver disease, when compared with non-diabetic patients with hepatic dysfunction [8].

The red blood cells (RBC) play an important role in vascular function, delivering oxygen, minimizing NO scavenging, and further delivering NO bioactivity in hypoxia, through the compartmentalization of hemoglobin (Hb); therefore, RBC promote hemostasis through a well-regulated delivery of oxygen [9]. In fact, reduced levels of NO derived from arginine has been implicated in the vascular dysfunction of diabetic patients. This pathological process is characterized by an impaired endothelial cell production of the vasodilator and an anti-platelet adhesion factor, and/or a decreased NO bioavailability [10].

An increased activity and expression of arginase I is associated with diabetes-induced increase in oxidative stress. It also initiates the feed-forward cycle of diminished NO levels and oxidative stress [11]. Moreover, citrulline could promote NO production and endothelial function and improve peripheral insulin sensitivity [12], improving organ perfusion and the endothelial metabolism, which might involve an antioxidant property [13].

In this context, we have found that type 2 DM alters the metabolite distribution between serum and RBC. Results demonstrate that RBC regulate serum levels of nitrogen-related metabolites, not only by their transport but also by metabolizing amino acids such as arginine, probably through several enzyme-mediated metabolic pathways [14]. In fact, this extra-hepatic nitrogen metabolism, mediated by blood cells, could have a role in the specific physiopathology of type 2 DM. These findings indeed agree with proteomic profiling studies which showed that the mature human RBC have a lot of proteins related to metabolic function, which reveals an unexpected level of complexity in the functional capabilities of human RBC metabolism [15].

Moreover, it has been reported that there are differences in the nitrogen metabolism in human subjects according to gender. Changes in vascular NO activity may contribute to changes in cardiovascular risk associated mainly with males, probably related to the α-adrenoceptor responsiveness, among other mechanisms [16]. The renal vasculature of men becomes more dependent on NO with age compared with that of women, suggesting that renal diseases progress could be related to gender [17]. Despite the fact that there is little information regarding the management of ammonia according to gender, it is noteworthy that hepatic myelopathy and blood ammonia elevation were found mainly among middle-aged Japanese men with chronic liver disease [18].

Based on the evidence above, we argue that patients with type 2 DM have an aggravated dysregulation of nitrogen metabolism and, therefore, the disturbed intermediary metabolism has more detrimental consequences in several organs. This altered nitrogen metabolism is influenced by gender and oxidant status. Consequently, this study improves our understanding of the relation between the disturbances in several metabolites reflecting nitrogen and nitric oxide (NO) metabolism, and abnormal levels of blood ammonia in the absence of evident nephropathy and liver disease.

## Subjects and methods

### Study population

We recruited patients with type 2 DM at different stages of the disease, from the outpatient clinics, mostly directly depending on the Ministry of Public Health (SSA, México), mainly from the Xoco General Hospital. The study group consisted of 40 female and 40 male patients with type 2 (non-insulin-dependent) DM. The patients were selected based upon the following criteria: All patients were not active alcoholics, non-smokers, and were on regular treatment for diabetes (with hypoglycemic agents). Patients were apparently free from any important secondary complications such as nephropathy or liver dysfunction. We studied in parallel a control group of healthy 40 female and 40 male who were non-smokers and non-alcoholics. Following a 12 h overnight fast, all individuals were subjected to blood sampling and all patients omitted their morning medication. This study was carried out in accordance with the Declaration of Helsinki (2000) of the World Medical Association and approved by the Ethics Committee of the General Hospital of Mexico (Ministry of Public Health), after written informed consent.

### Clinical tests

We measured in serum, through standardized procedures with kits from SPINREACT (Spain), the following metabolites: glucose, cholesterol, triacylglycerols (TG), and the activities of “marker enzymes” of liver damage, namely alanine (ALT-GPT) and aspartate (AST-GOT) aminotransferases. Insulin level was measured with a kit from Ray Bio (USA) and Hb A_1C_ was measured with a kit from BioSys-Kovalent (Brasil) (Table 1).

**Table 1:** Clinical parameters in control subjects and in female and male patients with type 2 DM.

Subjects	Healthy volunteers		Patients with type 2 DM	
Parameter	Females	Males	Females	Males
BMI	25.6	24.8	27.1	26.5
Glucose (mg %)	88	94	149	142
Insulin (μUnits/ml)	6.4	4.6	8.34	10.1
HOMA-IR	1.7	1.5	3.6	3.5
Hb A_1C_ (%)	4.6	5.0	8.8	8.4
Hb A_1C_ (mmols/mol)	27	29	73	70
TG (mg/dL)	141	148	185	175
Cholesterol (mg/dL)	150	138	185	179
ALT-GPT (IU/L)	18	21	22	23
AST-GOT (IU/L)	19	17	22	25

The results are expressed as medians. Abbreviations: BMI, body mass index; TG, triacylglycerols; ALT, alanine aminotransferase, and AST, aspartate aminotransferase. Most of the distributions were best described by skewed distributions, thus the mean is no longer a good measure of central tendency. Consequently, we relied on the median to compare between sets of data (S1 and S2 Files).

### Biochemical measurements

Whole blood was poured directly into ice-cold perchloric acid (8% w/v, final concentration). After centrifugation, we obtained acid-extracts of serum and RBC. We determined, using enzymatic methods, levels of ammonia [19] and free urea [20] in the neutralized perchloric extracts and assessed the uric acid using a commercial kit (SpinReact, Spain). In addition, we measured arginine, citrulline, ornithine, nitrites, and malondialdehyde (MDA), with the methods previously described in detail [14]. (dx.doi.org/10.17504/protocols.io.q5qdy5w).

### Preparation of RBC for metabolites release assays

Another set of anti-coagulated blood samples was obtained from healthy subjects (40 female and 40 male) and diabetic patients (40 female and 40 male). The serum was rapidly separated and removed after centrifugation at 900 g and 4°C for 5 min, the buffy coat was removed, and the erythrocyte pellet was washed four times with two volumes of cold (4°C) buffered solution of 20 mmol/L HEPES (pH = 7.42) and containing 0.9% NaCl. Thereafter, RBC were gently suspended to a 33% hematocrit (Hct) with the same buffered solution and stored at 4°C overnight, completing 24 h after washing. We obtained acid extracts from aliquots taken from all the washes, the overnight stored solution, and the remaining total RBC pellet. Then, we measured all the metabolites described above from them.

### Arginase activity in RBC

The activity of arginase (EC 3.5.3.1) was determined in RBC lysates with 20 mmol/L arginine at pH = 9.5, essentially as described by Colombo and Konarska [21]. The results are expressed as nmol of ornithine • min^-1^ • mg^-1^ of Hb.

### Calculations and statistical analysis

We calculated the concentration of serum and RBC metabolites as μmol • ml^-1^ or nmol • ml^-1^, and the results are expressed as the mean ± SD for levels of metabolites showed. **Statistical analysis**. We have a total of 84 determinations according to metabolite, blood compartment, group, and gender. Each of these data sets has 40 values. We took several blood samples for each patient and each person in the control group, and we obtained determinations from clusters of samples (pooled samples for each individual). Consequently, all results and conclusions drawn out from the analysis should consider that results correspond to the clusters and not to individual patient’s data. All data sets are available at the Data sets section of the open science framework project (see https://osf.io/a6cd3/?view_only=90dfd426c06648f5bbb96470c689d106).

The statistical analysis described in this section was performed in the Python 2.7 language using the numpy and scipy libraries (see Statistical analysis code in open science framework project: https://osf.io/a6cd3/?view_only=90dfd426c06648f5bbb96470c689d106). We computed the following descriptive statistics for each set of data: mean, standard deviation, skewness, and medians (**S1 File**). To study differences according to gender we focused on comparing medians since most of the data is best described by skewed distributions and are not normally distributed. In this situation, medians are a better measure of central tendency than means.

Furthermore, to analyze statistical differences according to gender, we first fitted the data to different distributions of continuous variables using maximum likelihood estimation and then obtained estimators for shape, location, and scale parameters for the distributions. We fitted the data to the following distributions: beta, exponential, exponential-weibull, exponential-power law, Gilbrat, logistic, lognormal, normal, Pareto, power law, weibull minimum, and weibull maximum. Then with the maximum likelihood estimators (MLEs), we performed the Kolmogorov-Smirnov (KS) goodness of fit test to obtain the distribution that best described the data for each determination. We chose the best approximation as the distribution which had the smallest D-statistic among all distributions. The KS test for goodness of fit performs a test of the distribution of an observed random variable against a given distribution under the null hypothesis that the two distributions are identical. We report D-statistics and p-values of the KS tests, and MLEs for each determination (**S2 File**).

Finally, we computed Spearman correlation coefficients and their p-values. We use the Spearman correlation because it assesses monotonic relations and it does not assume a linear relation between two sets of data. With the Spearman correlations, we constructed a correlation matrix for all 84 determinations (see **S3 File**). Each row and each column corresponds to a data set of one determination. The cells of the matrix are the Spearman correlation coefficient between two sets of data and the p-value. The Spearman correlation coefficient can take values between -1 and 1, where 0 means no correlation.

## Results

### Clinical parameters

Most of the distributions were best described by skewed distributions, thus the mean is no longer a good measure of central tendency. Consequently, we relied on the median to compare between sets of data. The medians obtained for the values of the distributions for some clinical parameters (**S1 File, Table 1**). The medians for blood glucose, insulin, HbA_1C_ and HOMA-IR, obtained for the control subjects are within the established “normal ranges”. In fact, insulin was lower in men, when compared to female healthy subjects. In the case of DM type 2 patients we observed a pronounced post fasting hyperglycemia (> 100 mg/dl), increased levels of Hb A_1C_ (>7%), and hyperinsulinemia (>8 μU/ml). These values are those expected for the two groups under study.

We performed the statistical analysis for each of these sets of values and compared the results. When comparing results for glucose between genders in the control subjects, we found that the best approximation shows different distributions (see **S2 File, Table 1**). The Spearman correlation coefficient was weak **S3 File, Table 1**).

In the case of patients with type 2 DM, the best approximation for glucose showed the same distribution in both genders, and interestingly, the same as in control men (logistic; **S2 File, Table 1**). Nevertheless, the Spearman correlation coefficient between genders for glucose in diabetic patients indicate that the correlation between male and female is low (0.1581; p-value 0.3296; **S3 File, Table 1**). Indeed, the comparison between Spearman correlation coefficient of glucose levels in control subjects and in diabetic patients, strongly suggest that together with the difference in glucose management between genders, there is a significant difference between the control subjects and the diabetic patients.

When we compared the values for insulin in the control groups of men and women, the Spearman correlation coefficient suggested a low weak negative correlation according to gender (-0.219; p- value 0.1742) (**S3 File, Table 1**). In the case of DM type 2 patients, the distributions of insulin were also different according to gender; these distributions were as well different compared to those observed for glucose in the same group. When comparing the Spearman correlation coefficient for insulin in the same gender, between control subjects and diabetics, we found that for both men and women, there is a null correlation, but in the case of women, the distribution is in the opposite direction (**S3 File, Table 1**). These differences may indicate that the mechanisms involved are connected to other systems that work differently between genders.

With respect to triglycerides, the best approximation for men and women, both for control subjects and for diabetic patients, shows different distributions (see **S2 File, Table 1**), but the Spearman correlation coefficients (**S3 File, Table 1**), indicate a low correlation between genders. These differences in distributions suggest differences in the production and regulation of triglyceride synthesis between genders and also between both study groups. As to cholesterol, the best approximation of values in both genders of control subjects and in diabetic men, showed the same distribution (see **S2 File, Table 1**). Here, the Spearman correlation coefficient indicated a low correlation in the distribution of cholesterol between women and men in control subjects (**S3 File, Table 1**). The distribution in diabetic women was different and the Spearman correlation coefficient between genders of diabetic patients was close to zero, indicating a null correlation (See **S3 File, Table 1**).

When comparing the Spearman correlation coefficient for cholesterol in the same gender, between control subjects and diabetic patients, we found that, for both men and women, there is a low correlation between the groups studied (**S3 File, Table 1**). In the case of women, the distribution is in the opposite direction compared with men, in a manner similar to the distribution of insulin, which suggests important differences in the interconnections between genders.

Results of the statistical analysis of the data obtained for the transaminases enzymes ALT and AST indicated that best approximation for both determinations was different between men and women in control subjects as well as in DM type 2 patients (see **S2 File, Table 1**). In control subjects the Spearman correlation coefficient suggests a weak and positive correlation according to gender for ALT and AST (**S3 File, Table 1**), whereas in the case of DM type 2 patients, the relation between genders is null for ALT and weak negative for AST. These results suggest a change in the behavior of activities of both enzymes as a result of pathology. The analysis of the results of the clinical parameters in DM type 2 patients could indicate differences in their production or utilization according to gender. In general, results maintain the differences between genders and also show different effects in the handling of glucose and cholesterol between control subjects and DM type 2 patients.

### Blood levels of ammonia, uric acid and urea in patients with type 2 DM

**Fig 1:**
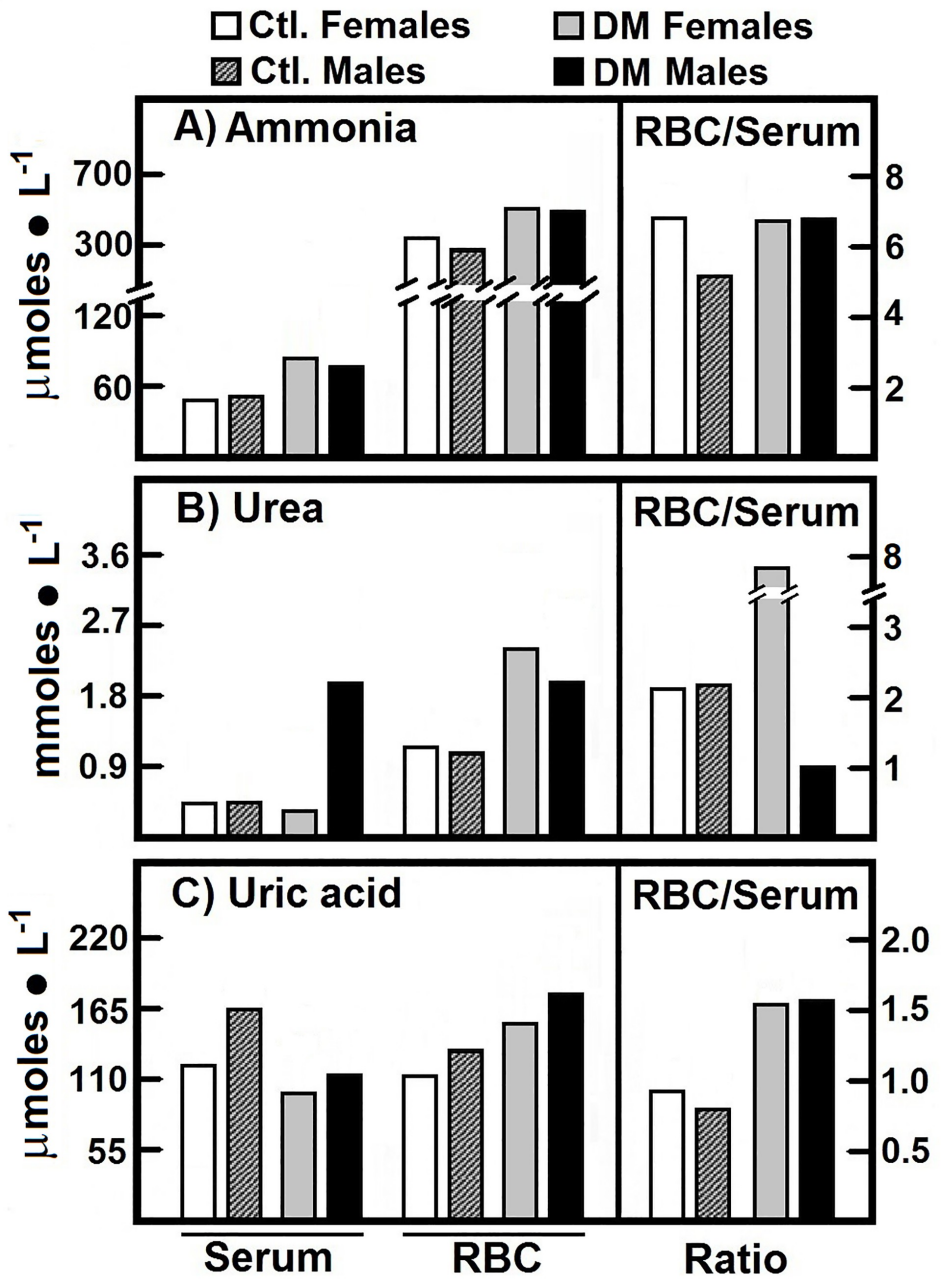
Metabolites participating in nitrogen metabolism quantified in serum and RBC from control subjects and in patients with type 2 DM. The results are expressed as the medians for levels of ammonia, urea, and uric acid, in healthy control female (n = 40) and male volunteers (n = 40), as well as in female patients with type 2 DM (n = 40) and male patients with type 2 DM (n = 40). Symbols for experimental groups at the top. Descriptive statistics: Most of the distributions were best described by skewed distributions, thus the mean is no longer a good measure of central tendency. Consequently, we relied on the median to compare between sets of data.

Ammonia, uric acid, and urea are products of the metabolism of proteins (nitrogen metabolism). The median values obtained for our control groups are those shown in the **Fig 1.** In the control groups, the “free” ammonia clearly predominated in the RBC over serum. Statistically, we found that the ammonia values for the control subjects are best described by different distributions according to gender and blood compartment (see **S2 File, Fig 1**). The Spearman correlation coefficients for RBC-ammonia of the control subjects indicated a null correlation between genders (**S3 File, Fig 1**).

We found an accumulation of ammonia in RBC as well as a drastic enhancement of serum ammonia, in female and male patients with type 2 DM. Nevertheless, the RBC/serum ratio for ammonia was similar in the four groups (**Fig 1A**). The Spearman correlation coefficients indicate a low positive correlation between diabetic female and male ammonia levels in RBC (**S3 File, Fig 1**). These results give evidence in support of the differential regulation of ammonia according to gender.

The blood urea levels, in all subjects were within normal values (< 3.33 μmol• ml^-1^). Urea predominated in the RBC fraction in a similar way to the ammonia levels. We noted that serum urea was lower in the male control group than the female control group, which led to an increased RBC/serum ratio of urea in the male control group (**Fig 1B**). The data obtained for the blood urea levels in the control groups showed different distributions depending on genders and blood compartment (**S2 File, Fig 1**). Here, we found a null correlation in RBC and a low positive correlation in serum levels (see **S3 File, Fig 1**), suggesting gender differences in blood urea levels in control subjects (**Fig 1B**).

The type 2DM patients had a drastic increase of urea in both blood compartments except for serum in women, also indicating clear gender differences for blood urea levels. Women patients had a significantly higher RBC/serum ratio for urea than the female controls, while DM male patients showed an opposite behavior (**Fig 1B**). These results show that in diabetic patients, RBCs accumulate both ammonia and urea, possibly to keep these metabolites in serum within normal levels, without gender differences. The statistical analysis pointed out of the data obtained for the urea in the DM type 2 patients (see **S2 File, Fig 1**). The Spearman correlation coefficient indicates a positive weak correlation between genders for urea levels in RBC and serum (see **S3 File, Fig 1**). These results, together with the above, provide strong evidence in favor of the differences in metabolic regulation according to gender.

The type 2 DM patients showed significantly lower serum levels for uric acid when compared to their respective controls, without modifying those in the RBC fraction. This resulted in higher RBC/serum ratios of uric acid in both genders with type 2 DM (**Fig 1C**). The statistical analysis for the uric acid determinations shows differences between genders. Nevertheless, uric acid in serum of control and diabetic males, and in RBC and serum of diabetic females, show the same distribution (see **S2 File, Fig 1**). The Spearman correlation coefficients indicate a positive low correlation between RBC values of control female and males, but a strong positive correlation between the control male and female serum values for uric acid (0.6439, p-value 0.0007). For DM type 2 patients the correlation was significant and positive in RBC (0.6247, p-value 0.0001) and positive but weak in serum (0.2407, p-value 0.1344) (**S3 File, Fig 1**).

We also measured arginase activity in RBC from control subjects and patients with type 2 DM. We did not find differences by gender in control subjects (2.17 vs. 2.62 nmol • min^-1^ • mg^-1^ of Hb, in women and men, respectively). On the contrary, in the DM type 2 patients indeed increased this enzyme activity. The female patients showed an activity of 3.84 nmol • min-1 • mg-1 of Hb, which was even higher in diabetic men (6.06 nmol • min-1 • mg-1 of Hb). These significant increases of arginase activity in RBC from patients with type 2 DM could be explain the increased RBC urea amount in these patients (**Fig 1B**).

Upon comparing the Spearman correlation coefficients between arginase in RBC and urea in serum, we observed that for both genders in DM type 2 patients, the Spearman correlation coefficients indicate a moderate correlation between arginase in RBC and serum urea, although in inverse senses (**S3 File, Fig 1**).

### Metabolites involved in the generation of NO in patients with type 2 DM

The arginine metabolism involving nitric oxide synthetase (NOS) results in the production of citrulline and NO [5]. We have shown that the human RBC in healthy subjects is capable to metabolize arginine into nitrites, citrulline, ornithine, and urea [14], we analyzed these metabolites in DM type 2 patients and compared the results. In the control group we observed that blood arginine was mostly present in RBC, compared to serum (**Table 2**). The statistical analysis of RBC-arginine values from control subjects showed that the behavior for both genders was best described by the same distribution (**S2 File, Table 2**), giving an evidence of a positive and significant relation between the values according to gender. Serum arginine levels in the same subjects were around 0.2 mmol • L^-1^ for both genders, and the Spearman correlation coefficient showed a positive and weak correlation (**S3 File, Table 2**), indicating differences according to gender and blood compartment.

**Table 2:** RBC and serum levels of nitrogen-related metabolites and their ratios in female and male patients with type 2 DM.

Subjects	Healthy volunteers		Patients with type 2 DM	
Parameter	Females	Males	Females	Males
Serum-Arginine	0.20	0.18	0.19	0.12
RBC-Arginine	0.47	0.77	0.89	0.67
RBC/serum ratio	2.9	4.3	4.7	5.6
Serum-Nitrites	0.010	0.017	0.056	0.040
RBC-Nitrites	0.094	0.117	0.074	0.068
RBC/serum ratio	9.4	6.9	1.3	1.7
Serum-Ornithine	0.062	0.064	0.060	0.069
RBC-Ornithine	0.127	0.176	0.207	0.177
RBC/serum ratio	2.0	2.8	3.5	2.6
Serum-Citrulline	0.233	0.245	0.606	0.577
RBC-Citrulline	0.179	0.151	0.364	0.311
RBC/serum ratio	0.77	0.62	0.60	0.54

The results are expressed as medians of the metabolites in μmol • ml^-1^. Most of the distributions were best described by skewed distributions, thus the mean is no longer a good measure of central tendency. Consequently, we relied on the median to compare between sets of data (S1 and S2 Files).

We found an arginine RBC/serum ratio of 4.3 for control male subjects and 2.9 for control female subjects (**Table 2**). In male patients with type 2 DM, there were not significant differences in serum arginine levels compared to RBC (**Table 2**). On the opposite, women with DM had higher levels of RBC-arginine than male patients, with congruently higher RBC/serum ratio for this amino acid, when compared to the control group (**Table 2**).

The medians of the nitrites determinations show that levels in RBC in control subjects and diabetic patients were higher than in serum. We also found that nitrites determinations in RBC and serum of control male subjects were higher than those of control female. On the contrary in patients with DM type 2, the women showed an upper levels of RBC-nitrites (**S1 File, Table 2**). These results give evidence that type 2 DM reverted gender differences found for the nitrites blood distribution observed in the control groups (**Table 2**).

The statistical analysis of the nitrites determinations in control subjects shows that in the case of serum, the same distributions were obtained for control women and diabetic men. The distributions of the nitrites levels from RBC in control men and RBC and serum of diabetic women were also the same (**S2 File, Table 2**). There were positive weak and low correlations of values in RBC and in serum, respectively, between the both gender in control subjects. However, whereas that the Spearman correlation coefficients indicate a low correlation between levels of nitrites in RBC from female and male DM type 2 patients, (**S3 File, Table 2**), importantly a strong and positive correlation was obtained between levels of nitrites for diabetic men and women in serum (**S3 File, Table 2**). As previously found for other metabolites, the statistical distributions and the Spearman correlation coefficients indicate differences in nitrites levels according to gender and blood compartments.

Arginine is synthesized from citrulline by the sequential activities of the cytosolic enzymes argininosuccinate synthetase (ASS) and argininosuccinate lyase (ASL), and these proteins have already been identified in RBC [22]. While serum levels of ornithine showed no differences according to gender, the male control group showed higher RBC-ornithine than the control females (see **S1 File, Table 2**). Type 2 DM promoted higher ornithine content in RBC of female patients but not of men. This led to a significant increase the RBC/serum ratio for ornithine in the female patients, in contrast to the gender differences found in healthy subjects (**Table 2**).

On the contrary, blood citrulline was mainly found in serum, resulting in an RBC/serum ratio for citrulline below one. Here, control men presented a significantly lower level of RBC-citrulline than the healthy female group (**Table 2**). The onset of type 2 DM elicited an increase of citrulline in both blood compartments but maintained the RBC/serum ratios for this amino acid. Consequently, this pathology also annulled gender differences in the citrulline blood levels observed in control subjects (**Table 2**).

### Relations between blood metabolites in serum and RBC in patients with type 2 DM

The aforementioned results allowed us to shed light on the possible metabolic paths followed in serum and RBC. The arginine/nitrites and citrulline/nitrites relations in serum were higher in healthy women compared to men (**Table 3**). However, the group of female patients with type 2 DM showed altered relations of these nitrogen compounds when compared to healthy women. The relations in serum of ammonia/urea, arginine/urea, and citrulline/ornithine were significantly higher, while that of arginine/ornithine did not change. On the contrary, the other relationships shown in **Table 3** were found significantly lower in women with type 2 DM, when compared to the healthy female group. With respect to men, although the ammonia/urea was lower in men with type 2 DM, the other serum relationships among nitrogen compounds had the same pattern of behavior as that of type 2 DM women. However, the differences were even larger in men (**Table 3**).

**Table 3:** Nitrogen-related metabolites and their ratios in serum and RBC of patients with type 2 DM.

Subjects	Healthy volunteers		Patients with type 2 DM	
Serum ratios	Females	Males	Females	Males
Ammomia/urea	0.12	0.13	0.24	0.04
Arginine/ammonia	4.1	3.6	2.4	1.6
Arginine/urea	0.49	0.47	0.58	0.06
Arginine/nitrites	20.0	10.6	3.2	3.0
Arginine/ornithine	3.2	2.8	3.2	1.7
Arginine/citrulline	0.86	0.73	0.31	0.21
Citrulline/nitrites	23.3	14.4	10.8	14.4
Citrulline/ornithine	3.8	3.8	10.1	8.4
RBC ratios	Females	Males	Females	Males
Ammomia/urea	0.29	0.24	0.22	0.26
Arginine/ammonia	11.7	23.9	14.0	10.9
Arginine/urea	3.5	5.8	3.0	2.8
Arginine/nitrites	41.9	59.3	99.1	82.4
Arginine/ornithine	31.0	36.5	35.4	31.6
Arginine/citrulline	22.0	42.5	20.1	18.0
Citrulline/nitrites	1.9	1.3	4.9	4.6
Citrulline/ornithine	1.4	0.9	1.8	1.8

Descriptive statistics. We computed the arithmetic mean, the variance, the skewness, and median for each determination. Most of the distributions were best described by skewed distributions, thus the mean is no longer a good measure of central tendency. Consequently, we relied on the median to compare between sets of data.

The relations in RBC were significantly different according to the gender (**Table 3**). In this blood compartment, the relations arginine/ammonia, arginine/nitrites, and arginine/citrulline, were also higher in control men than those found in the healthy female group. On the contrary, the citrulline/nitrites as well as the citrulline/ornithine relations were lower in the male control group than in the healthy women (**Table 3**). These results, when related to the pattern of relationships in serum of control subjects, argue in favor of gender differences in the “management” of ammonia and nitrogen-related compound (**Table 3**). In addition, with the exception of the relationships of ammonia/urea and arginine/ammonia, the onset of type 2 DM in male patients readily neutralized gender differences in the blood distribution of these metabolites, seen in the healthy volunteers (**Table 3**).

### Release of ammonia, urea, and arginine from RBC of patients with type 2 DM after washing and storage

**Fig 2:**
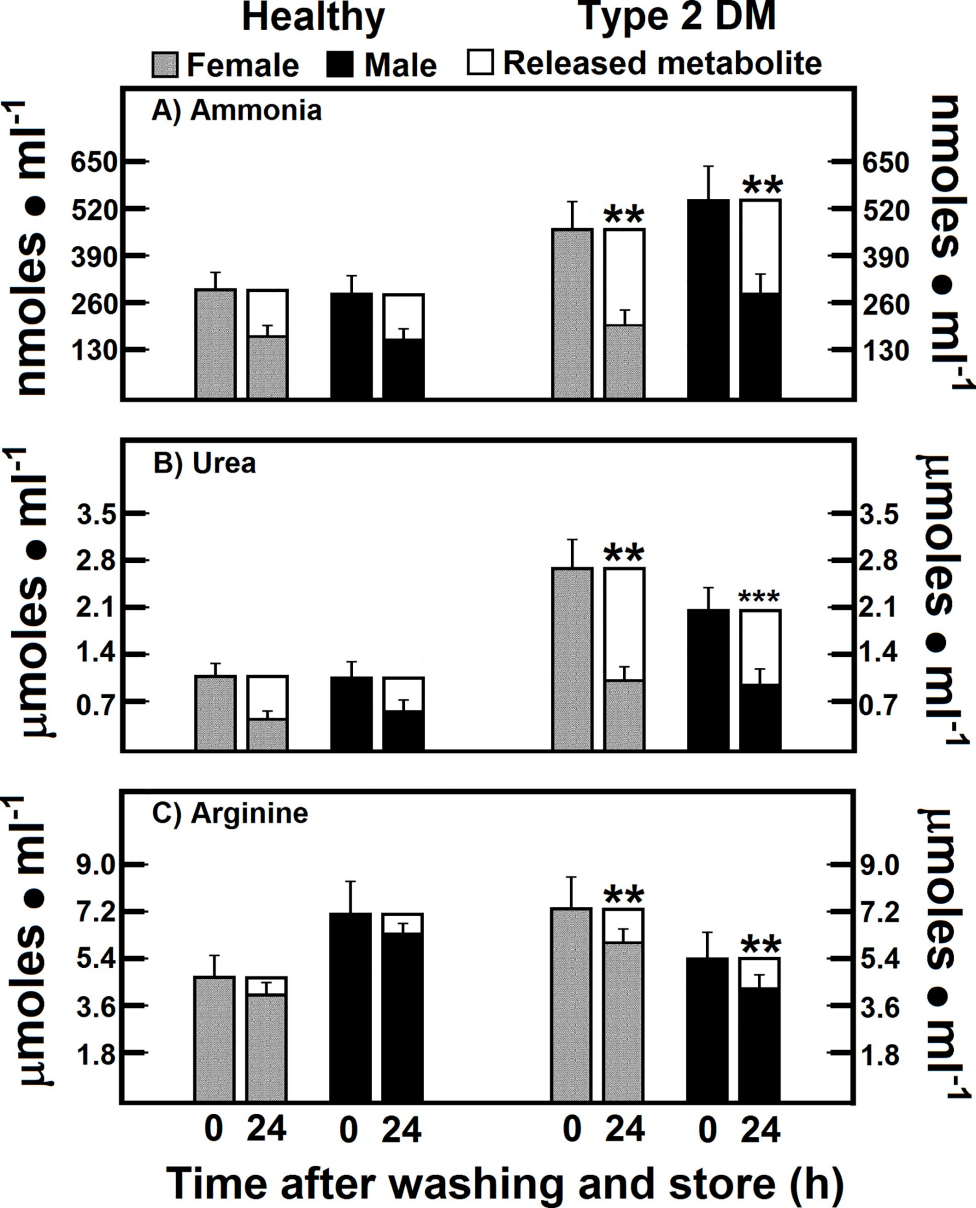
Release of ammonia, urea, and arginine from RBC of patients with type 2 DM after washing and storage. The results are expressed as the mean ± SD for levels of RBC ammonia, urea, and arginine, in healthy control female (n = 40) and male volunteers (n = 40), as well as in female (n = 40) and male DM type 2 patients (n = 40), at time 0. Empty bars represent the release rate for each metabolite 24 h after washing and storage (time 24). Symbols for experimental groups at the top. Statistical significance: *p < 0.01 against the control female group, and **p < 0.01 vs. the corresponding gender control group.

Gender differences in amino acid concentrations in serum have been recorded in normal healthy Japanese people [23]. Therefore, to make an effort to explain the gender differences in healthy subjects and patients with DM we measured the metabolite “release” from the RBC of both experimental groups. The RBC from control female and male subjects showed an efflux of ammonia of 38.2% and 44.5% of total RBC content, respectively, after blood cells washing and storage (**Fig 2A**). In this context, patients with DM released even more ammonia (56.9% and 47.6% in women and men, respectively), which resulted in having a similar amount of ammonia, when compared to healthy subjects (**Fig 2A**).

Interestingly, RBC from healthy subjects had an efflux of urea of 58.4% and 47.7% of total RBC content in women and men, respectively. The patients with type 2 DM released a quite similar amount of urea than healthy subjects (63.0% and 53.9% in women and men, respectively, of the total RBC content) (**Fig 2B**). The RBC content of arginine showed a very small release of 11.5% and 11.1% of total RBC content in control women and men, respectively (**Fig 2C**). RBC from the patients with DM almost doubled the release of arginine (19.4 and 20.8% of total RBC content in women and men, respectively), even though the remaining RBC levels for arginine (**Fig 2C**) were almost the same as those found in non-washed blood cells, mainly in control preparations (**Table 2**).

### Release of nitrites, ornithine, and citrulline from RBC of patients with type 2 DM after washing and store

**Fig 3:**
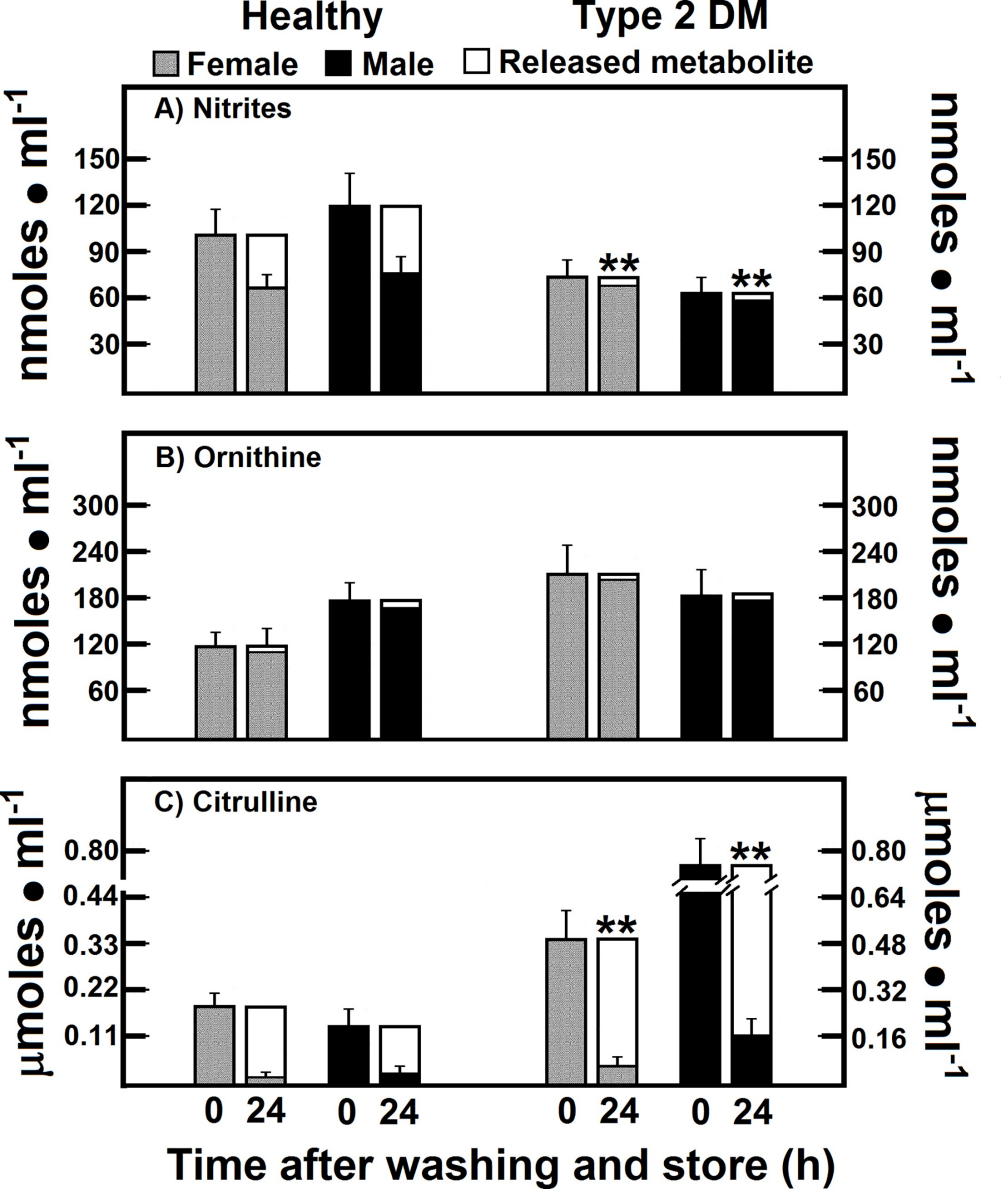
Release of nitrites, ornithine, and citrulline from RBC of patients with type 2 DM after washing and storage. The results are expressed as the mean ± SD for levels of RBC nitrites, ornithine, and citrulline, in healthy control female (n = 40) and male volunteers (n = 40), as well as in females (n = 40) and males (n = 40) with type 2 DM at time 0. Empty bars represent the release rate for each metabolite 24 h after washing and store (Time 24). Symbols for experimental groups at the top. Statistical significance as pointed out in the **Fig 2**.

The RBC content of nitrites, which are NO oxidation products, also showed changes after washing and storing the blood cells (**Fig 3A**). The efflux of nitrites from RBC in controls after washing and storing was 34.3% and 37.9% of total RBC content in women and men, respectively. On the contrary, RBC in patients with type 2 DM showed a lower release of nitrites (less than 10% of total RBC content) in both experimental groups **(Fig 3A),** which contrasted with the high levels of serum nitrites found in both, women and men DM patients (**Table 2**).

The efflux of ornithine in the RBC from DM patients, was closely similar to that of control cells after washing and storage (**Fig 3B**). We conclude that RBC do not release ornithine, since the rate of release of this amino acid was lower than 6% (**Fig 3B**). Interestingly, the citrulline that differs very little chemically from ornithine was readily released after washing and storage (more than 90% of the initial value) in female or male control subjects (**Fig 3C**). RBC-citrulline in type 2 DM patients also showed a reliable efflux under our experimental conditions; however, this release was proportionally lower in type 2 DM patients. Nevertheless, we also observed a gender difference, as men with type 2 DM retained more citrulline in their RBC than women (**Fig 3C**). Indeed, present data confirm the existence of a net efflux of by-products of RBC arginine metabolism in RBC of control and patients with type 2 DM, determined by radio-labeled tracing as previously reported under these experimental conditions [14].

### Correlation between levels of ammonia and MDA and their release from RBC in patients with type 2 DM

**Fig 4:**
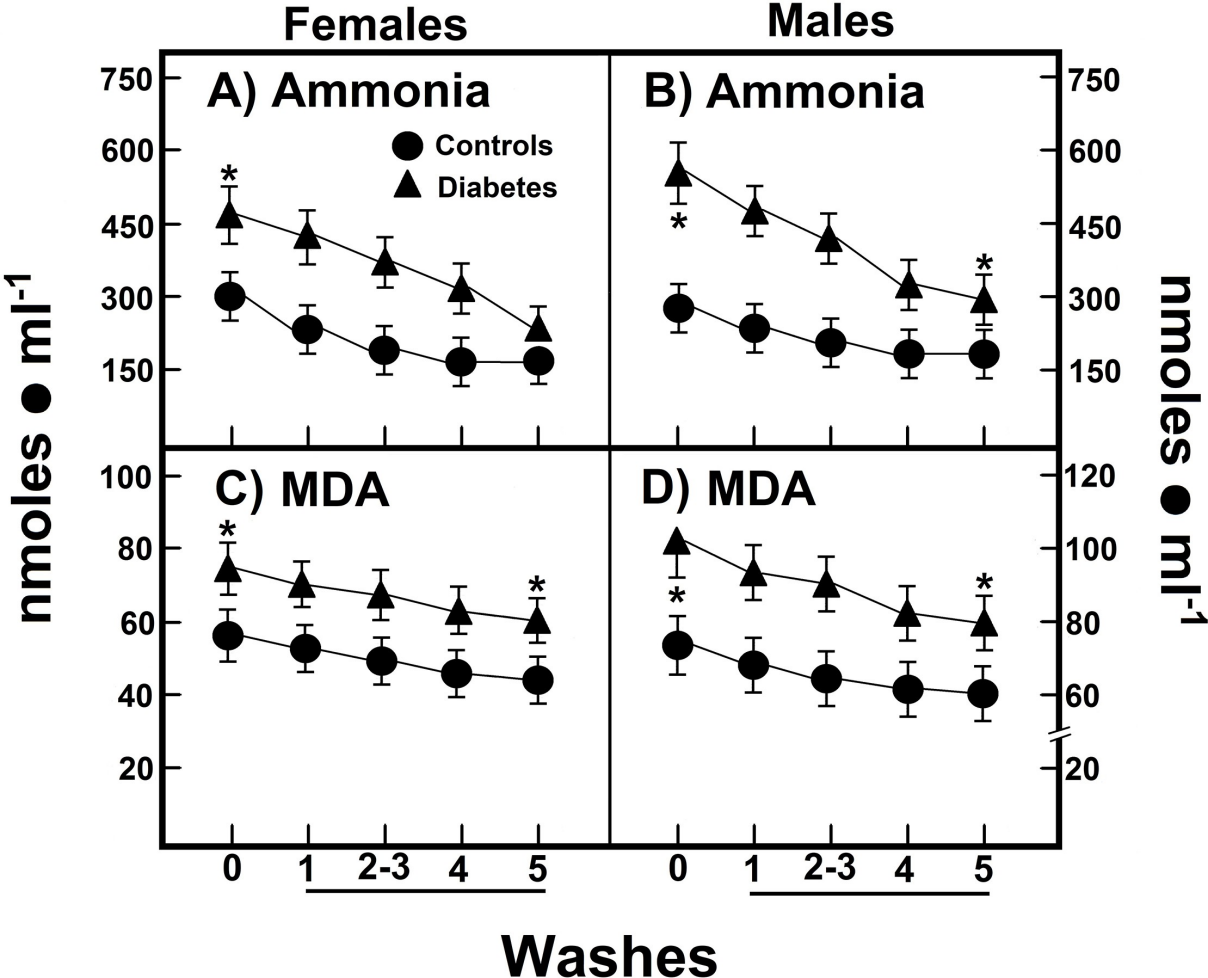
RBC amount and curves of release of ammonia and MDA from RBC of patients with type 2 DM after washing and storage. The results are expressed as the mean ± SD for levels of RBC ammonia (panels A and B), as well as of malondialdehyde (MDA; panels C and D), in healthy control female (n = 40) and male volunteers (n = 40), as well as in females (n = 40) and males (n = 40) with type 2 DM. The wash numbers 5 correspond to storage of RBC in the washing solution for 24 h. Symbols for experimental groups at the top of the figure, and statistical significance as indicated in the Fig 2.

For this section, we used the Pearson correlation coefficient and the mean and standard deviations to report results. When we calculated the area under the curve (AUC) for the progressive release of this ammonia from RBC of female subjects (6.4 ± 1.9 nmol • h^-1^ • ml^-1^ of RBC), this value was not statistically different when compared to male volunteers (AUC = 5.2 ± 1.6 nmol • h^-1^ • ml^-1^ of RBC; **Fig 4A and 4B**). Female patients with type 2 DM showed a significant increase of RBC ammonia release (AUC = 9.9 ± 2.5 nmol • h^-1^ • ml^-1^ of RBC) when compared with healthy women (**Fig 4A and 4B)**. Interestingly, we also found a larger liberation of ammonia by RBC from diabetic male patients (AUC = 10.5 ± 2.9 nmol • h^-1^ • ml^-1^ of RBC), which is twice that of found in normal male subjects.

In women with type 2 DM the released RBC amount of MDA, a byproduct of membrane’s lipid peroxidation (AUC = 0.55 ± 0.15 nmol • h^-1^ • ml^-1^ of RBC) was not significantly different from that found in healthy women (AUC = 0.58 ± 0.17 nmol • h^-1^ • ml^-1^ of RBC; **Fig 4C**). On the contrary, male patients with type 2 DM released more MDA from their isolated RBC when compared with healthy men (AUC = 0.60 ± 0.16 vs. 0.86 ± 0.25 nmol • h^-1^ • ml^-1^ of RBC, in diabetic patients; **Fig 4D**).

### Correlations among nitrogen metabolites with serum glucose in patients with type 2 DM

In an attempt to define the specificity of the changes in blood nitrogen metabolism as a consequence of the alterations found in patients with type 2 DM, we investigated correlations among the different metabolites included in our experiments.

**Fig 5:**
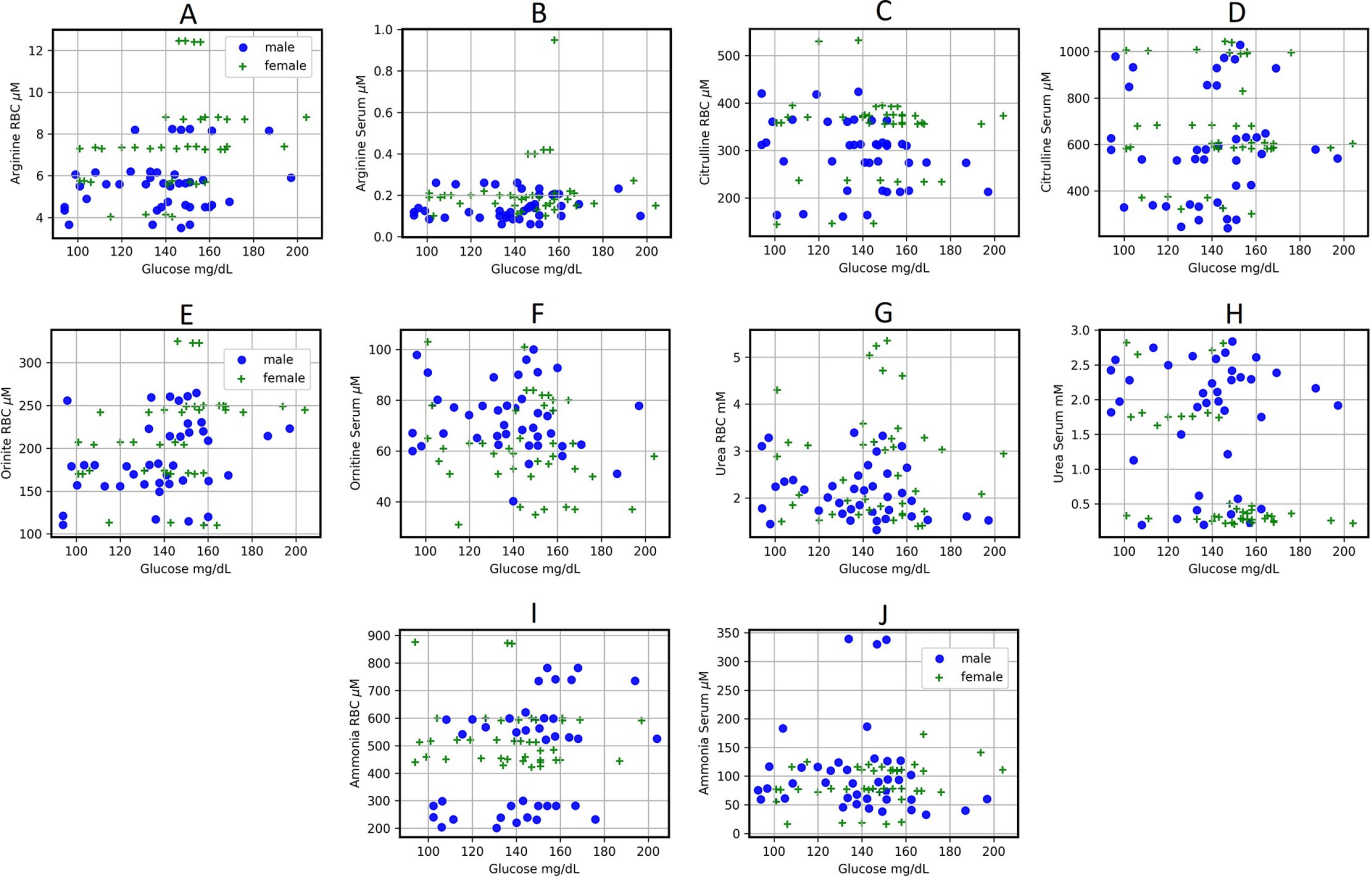
**Scatter plots for different determinations according to gender (males are circles and females are stars) and RBC (A,C,E,G and I) or serum (B,D,F,H, and J) in DM type 2 patients.** Scatter plots show the relation between two sets of data. This relation is summarized in the Spearman correlation coefficient (r) for each relation in each scatter plot:: A (rM = 0.08, rF = 0.39), B (rM = 0.14, rF = -0.014), C (rM = -0.301, rF = -0.086), D (rM = 0.113, rF = -0.044), E (rM = 0.299, rF = 0.425), F (rM = -0.133, rF = -0.163), G (rM = -0.227, rF = -0.04), H (rM = -0.013, rF = -0.559), I (rM = -0.132, rF = 0.322), and J (rM = -0.257, rF = 0.235). To see the p-values for each correlation, see the correlation matrix (S3 File).

There was a null Spearman correlation coefficient between RBC-arginine and glucose in male diabetics, indicating that these metabolites are not related in this blood compartment. When analyzing the correlations between arginine and glucose in serum we observed otherwise, the diabetic women have a null correlation coefficient (0.0146, p-value 0.9286), whereas the diabetic men show a positive and low Spearman correlation coefficient (0.1420, p-value 0.3818; **Fig 5B**).

In diabetic males, the RBC-citrulline is inversely related with glucose, as evident from a weak negative Spearman correlation coefficient (**Fig 5C**). Also interestingly, serum levels of citrulline correlated directly with those of glucose in this patients with a low positive correlation for serum citrulline and glucose (**Fig 5D**). However, these correlations are lost in the case of diabetic women (**Fig 5C and 5D**).

The RBC levels of ornithine correlated weakly positive with serum glucose in both diabetic genders, although the Spearman correlation coefficient is higher in diabetic women **(S3 File, Fig 5E)**. Conversely, Spearman correlation coefficient suggests a low negative relation between serum ornithine and glycaemia in both diabetic genders (**S3 File**, **Fig 5F**).

We found that when the glucose serum levels increase, the levels of urea decrease in RBC and serum in both genders. The Spearman correlation coefficient between urea in RBC and glucose in serum is low from diabetic men and females (**Fig 5G**). Interestingly, the comportment is opposite when we compared according to gender both compounds in serum (**S3 File**, **Fig 5H**). The RBC and serum levels of ammonia had a low and inverse correlation with serum glucose in diabetic males (RBC: -0.132, p-value 0.1783; serum: -0.257, 0.1081. **S3 File, Fig 5**), while RBC and serum-ammonia had also a low but direct correlated with hyperglycemia in female patients with type 2 DM (**S3 File**, **Fig 5I and 5J**).

### Correlations between serum nitrites with ammonia and urea in patients with type 2 DM

It has been speculated that facultative anaerobic bacteria might reduce nitrite to NO and possibly further to form ammonia [24]. Therefore, we searched relationships among the circulating amounts of nitrites, arginine, urea, and ammonia.

**Fig 6:**
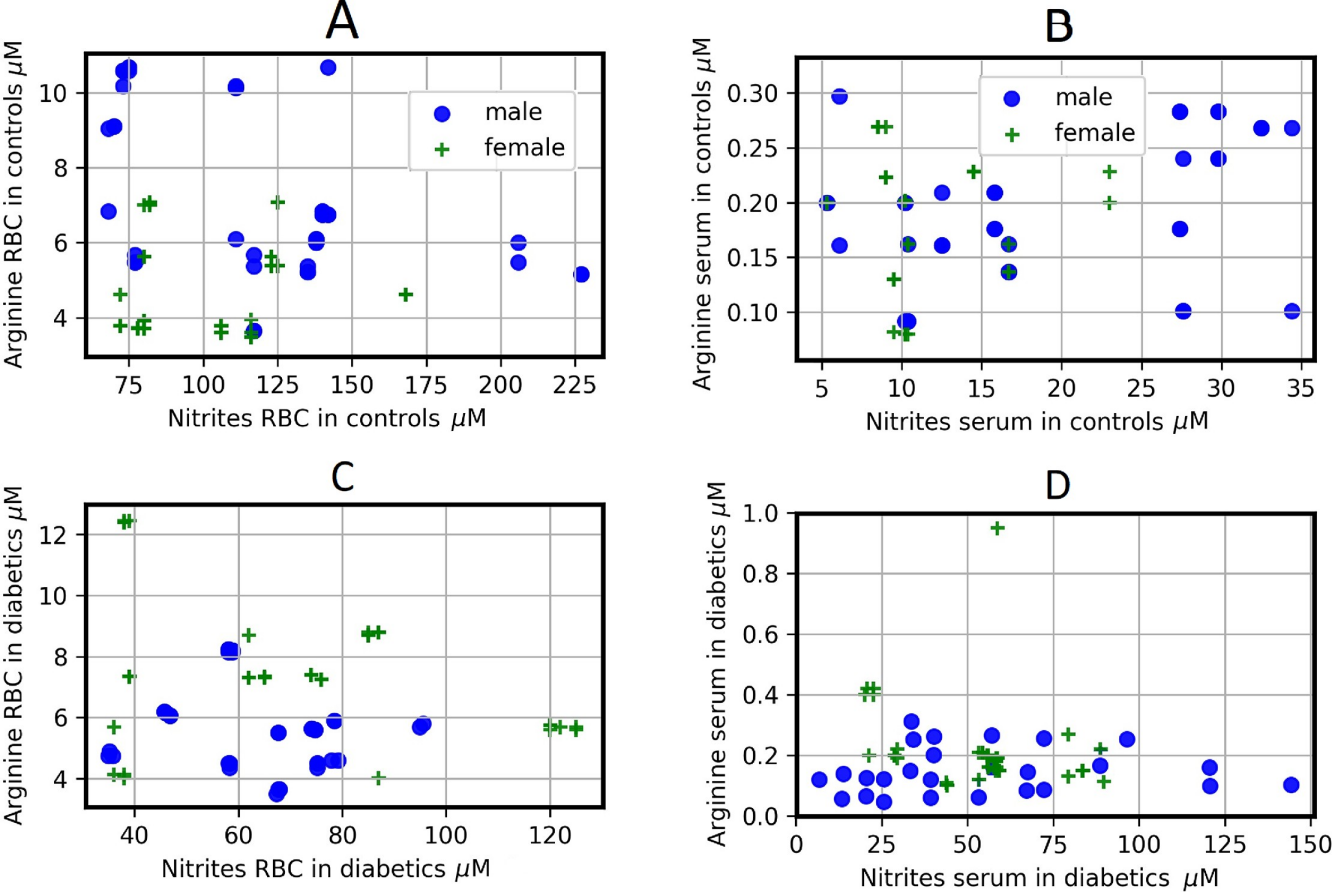
**Scatter plots for different determinations for controls and diabetic patients according to gender (males are circles and females are stars) and RBC (left column) or serum (right column).** Scatter plots show the relation between two sets of data; this relation is summarized in the Spearman correlation coefficients. These correlations are: A (rM = -0.439, rF = 0.269), B (rM = 0.276, rF = -0.22), C (rM = -0.2578, rF = -0.179), D (rM = 0.229, rF = -0.361). To see the p-values for each correlation, see the correlation matrix (S3 File).

In relation to gender, the Spearman correlations coefficients show that the correlations between arginine and nitrites have an opposite behavior in control subjects, both in RBC and in serum. A weak negative correlation can be observed between arginine and nitrites in RBC of control men), whereas in RBC of control women the Spearman correlation coefficient indicates a weak positive correlation (**S3 File**). In the case of serum, correlation between nitrites and arginine showed an opposite pattern (**S3 File, Fig 6A and 6B**).

**Fig 7:**
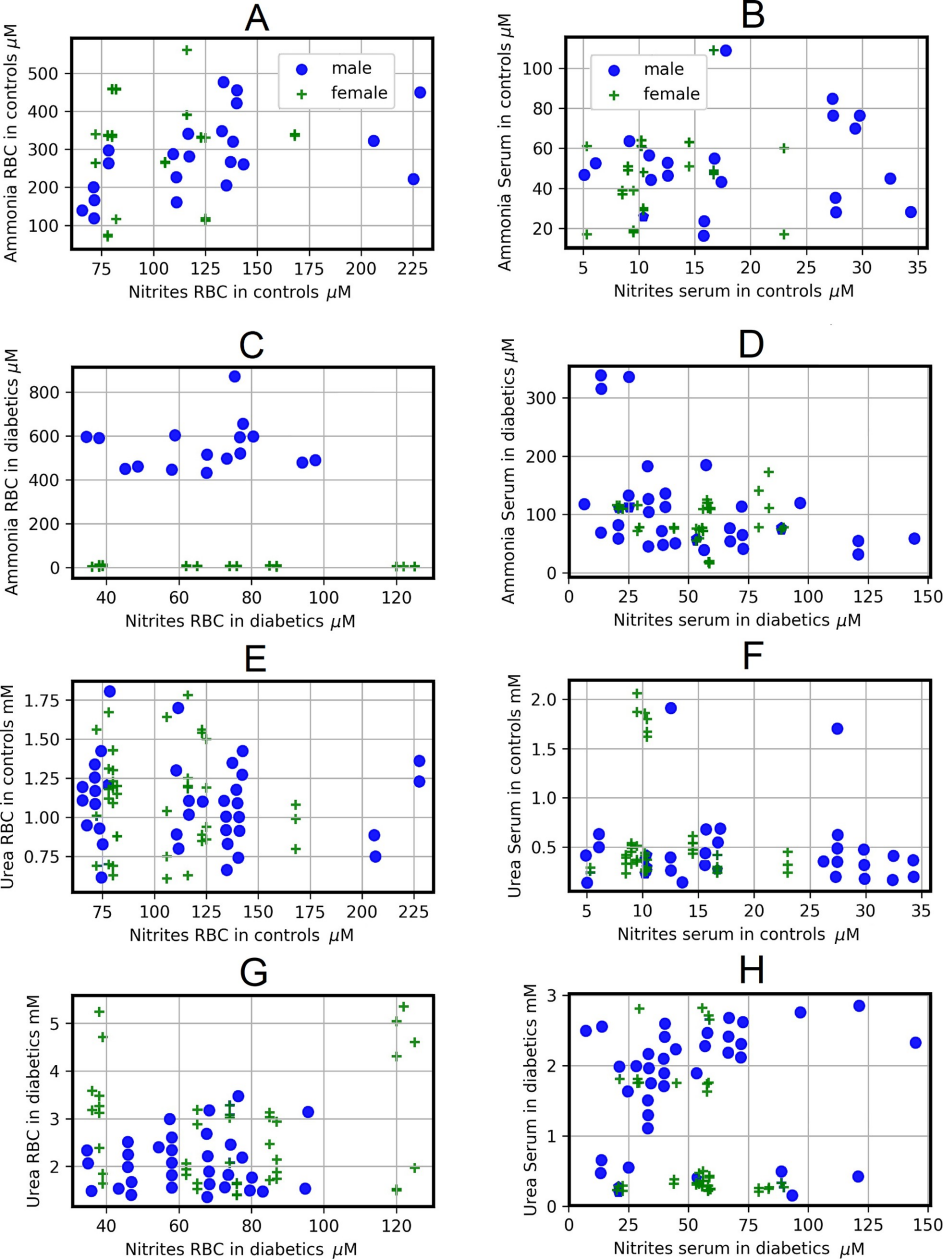
**Scatter plots for different determinations for controls and diabetic patients according to gender (males are circles and females are stars) and RBC (left column) or serum (right column).** Scatter plots show the relation between two sets of data; this relation is summarized in the Spearman correlation coefficients. These correlations are: A (rM = 0.562, rF = -0.0496), B (rM = -0.0388, rF = 0.3011), C (rM = 0.317, rF = -0.0588), D (rM = -0.4465, rF = -0.047). E (rM = -0.096, rF = -0.0636), F (rM = 0.0018, rF = -0.048), G (rM = -0.031, rF = - 0.1318), and H (rM = 0.3665, rF = -0.2506). To see the p-values for each correlation, see the correlation matrix (S3 File).

In serum of patients with DM type 2, the Spearman correlations coefficients between arginine and nitrites, showed a weak and inverse correlation pattern, while in RBC both correlations are weak and negative (**S3 File**, **Fig 6C and 6D**). If we compare the Spearman correlations coefficients between the levels of arginine and nitrites in both genders of control subjects with those of patients with type 2 DM, we observed that the pathology does not change the behavior of the correlations, neither in serum nor in RBC, with the exception of RBC of women, where the Spearman correlation coefficient goes from weak positive in control females to low negative in diabetics females (**S3 File, Fig 6**). Regarding the correlations between ammonia and nitrite, when comparing the Spearman correlations coefficients between RBC of control subjects and patients with type 2 DM of both genders, the pathology does not change the way in which they correlate (**Fig 7A and 7C**). Importantly, in serum the Spearman correlations coefficients between ammonia and nitrites change from null in control men to weak negative in diabetic men, whereas an inverse behavior is observed in women (**Fig 7B and 7D**). Control women have a weak positive correlation between ammonium and nitrites (**S3 File, Fig 7**), while DM type 2 women have a null correlation in this relation. Once again, the comparison of the Spearman correlations coefficients between genders shows us a different behavior in the correlations in both the groups studied.

Whereas we find null correlations between RBC or serum levels of nitrites and urea in control subjects (**S3 File**, **Fig 7E and 7F**), patients with DM type 2 exhibit different parameters. For instance, female patients had a low negative correlation between RBC-nitrites and those levels for urea (**S3 File**, **Fig 7G**), while in serum, the levels of nitrites and urea, both in male and in female patients with type 2 DM, showed weak correlation in these metabolites (**Fig 7H**). Therefore, results from **Figs 5, 6 and 7** strongly support the existence of a differential blood metabolism for nitrogen-related compounds by gender, which is largely disturbed by the presence of type 2 DM.

## Discussion

The RBC are the most abundant cell in humans and their alterations are deeply involved in health and disease. Indeed, as a result of the metabolomic and proteomic analysis, it has been understood that they are very important in the physiology of the body; close to 2000 gene products have been reported [25]. Moreover, to our knowledge, this study provides the first documented evidence of a putative role of blood cells (mainly attributed to RBC) in the control of serum levels of diverse metabolites, such as those related to the nitrogen metabolism in humans, which is largely altered by pathologies such as type 2 DM. We highlight two main findings. Firstly, the distribution of these metabolites in human blood compartments (serum and RBC) is influenced by gender, probably affecting bi-directional transport through RBC membranes and its metabolism [14]. Secondly, onset of type 2 DM abrogates many of the gender-related differences found in healthy subjects.

The hyperglycemia and the increase in levels of HbA_1C_ found in patients with DM were closely associated with blood levels for either, ammonia or urea (**Fig 5**). These results suggested that abnormal levels of blood glucose might induce alterations in the nitrogen metabolism. The latter can be related to the fact that RBC may undergo suicidal death or eryptosis, which is characterized by cell shrinkage and cell membrane alterations triggered by oxidative stress [26]. Exposure of normal erythrocytes to high glucose concentrations *in vitro* intensified lipid peroxidation (LP) and the loss of RBC enzyme activities [27]. Moreover, high levels of HbA_1c_ are closely related to LP in RBC, which are also associated to a significant reduction in membrane Na+/K+-ATPase activity [28,29].

Type 2 DM is characterized by low-grade, chronic inflammation and an increased activity of IkB/NFkB seems to provide a molecular mechanism responsible for inflammation and insulin resistance in type 2 DM, associated with increased risk of cardiovascular diseases [30]. In endothelial cells, excess free fatty acids activate the pro-inflammatory IKKβ–NF-κB pathway causing cellular insulin resistance and impaired NO production [31]. In this scenario, structural and/or functional alterations of RBC could be playing a role in the endothelial dysfunction noticed in patients with type 2 DM.

Studies have suggested that when the liver capacity for removing ammonia is reduced, other organs interact to maintain ammonia levels [32]. Glutamine functions as a “trapper” for excess ammonia, through the reaction of glutamine synthetase, and this occurs in the skeletal muscle [33]. Indeed, blood patterns for these amino acids should be altered in patients with chronic liver damage [34]; but, what about the alterations in the nitrogen metabolism found in female and male patients in absence of liver failure?

The end-products of the nitrogen metabolism, ammonia and urea, were greatly elevated in blood in diabetic patients (**Fig 1, Table 2**). Urea drastically augmented in RBC, which could be accounted for by the blood cells capacity to uptake and transport these metabolites (**Fig 1, Table 2**). Despite the apparent high production of urea by the liver of DM patients, ammonia definitively increased. Increased serum ammonia and glutamic acid levels found in patients with type 2 DM could be associated with delayed gastrointestinal transit [35], as well as with the increased activity of monoamine oxidase DB [36]. Since the presence of high blood levels for ammonia was not related to liver dysfunction, this could indicate that other extra-hepatic cells, such as RBC, are participating in the control of nitrogen metabolism [25].

Ammonia toxicity may induce cell damage, as it occurs during neuro-degeneration in aging, and Alzheimer disease [37] and, in diabetic rats there was a marked increase in the Na+-dependent component of the L-glutamate transport, causing a higher intracellular concentration of glutamate [38]. Whether the same occurs within blood cells remains to be studied; however, our findings provide evidence that RBC from the patients with type 2 DM can transport potential toxic nitrogen-related molecules (ammonia, urea, glutamic acid), which constitutes an unexplored risk factor for further complications. In experimental models for DM, a differential susceptibility for the harmful effects of ammonia on female or male animals has been reported. A porta-caval shunt reduces growth and spontaneous motor activity in male but not in female rats [39].

On the other hand, an intra-portal load of L-glutamine in rat with experimentally-induced type 1 DM generates increased glucose output, accompanied by enhanced urea and ammonia production [40]. These might be providing a background to explain the enhanced blood levels for ammonia and urea found in our patients with type 2 DM (**Figs 1, 2, 4 and 5**). In this context, we have recently shown an association between oxidant stress and elevated levels of ammonia [41], where increased systemic free ammonia concentration was capable of mediating the deleterious effects of AZT on partial hepactectomy-Induced rat liver regeneration. Indeed, the AZT significantly increased blood levels of ammonia and of MDA; despite this nucleoside did not reduce the amount of urea in the whole blood, but rather did change its distribution in the blood compartments [41].

Hyperglycemia is associated with increasing levels of six amino acids (alanine, isoleucine, leucine, valine, phenylalanine, and tyrosine) and with decreasing levels of histidine and glutamine; these associations were explained by insulin sensitivity [42]. Plasma branched chain and aromatic amino acids have been also associated with incident diabetes and underlying metabolic abnormalities [43], which could be a focus for identifying novel etiological mechanisms and treatment targets for DM.

An excess of ammonia can alter NO production; type 2 DM patients showed increased serum nitrites levels, as well as those of citrulline in both blood compartments, associated with a decreased blood arginine level, mainly observed in men with type 2 DM (**Table 2**). Interestingly, the existence of gender differences in the NO metabolism is currently known. Systemic NO production is higher in healthy premenopausal women than in men, paired by age, under ambulatory conditions. This variance could explain the differences in endothelial production of NO, and the differential vascular function observed in men and women [16,44]. Indeed, estrogens can play a role in modifying NO response in women. Estrogens can inhibit Ca^2+^ influx mediated by L-type Ca^2+^ channels, representing one way through which estrogen protects ischemic hearts [45].

The present study gives evidence that in healthy subjects exist significant gender differences in NO production and, for the first time, in arginine metabolism (**Tables 2 and 3, S2 and S3 Files**). The arginine/nitrites and arginine/citrulline ratios were bigger in RBC of healthy men than in women, suggesting a lower flow of arginine through putative nitric oxide synthase in men, and healthy men also seemed to use less arginine as substrate for arginase, i.e., the arginine/urea ratio (**Table 3**). These data agree with the aforementioned speculation that women seem to have a more active NO metabolism than men. Interestingly, serum arginine levels were maintained within a normal range in patients with DM; hence, the importance of maintaining normal serum arginine levels could be is related to its metabolic role as the ammonia detoxification product, and to production of L-citrulline and NO [5,46], improving RBC function and extending cell life span in old RBC [47]. So, it was noteworthy to find that onset of type 2 DM practically abrogates gender differences in nitrogen-related metabolites which are observed in controls (**S2 and S3 Files, Tables 2 and 3**).

We found significant relationships between serum nitrites levels with those of ammonia and urea, indicating the participation of NO metabolism in the management of blood ammonia and of urea (**Fig 6**). As to citrulline, what could cause its elevation in blood of patients with type 2 DM? Citrulline has beneficial effects such as the increased NO production and endothelial function through its conversion to arginine [48]. Therefore, it is attractive to speculate whether the massive outflow of citrulline from RBC is an attempt to reestablish NO metabolism, functioning as an antioxidant in RBC of type 2 DM patients.

Which mechanism is most probably involved in these unexpected results is left to be answered in future research. One possible explanation could be given by the role of RBC in handling molecules participating in the nitrogen metabolic pathways and the fact that these blood cells are altered by the occurrence of DM [14]. Indeed, we have shown that the RBC capacity for metabolizing arginine depends on the oxidant status and the lipid composition of RBC membranes, which are altered in patients with type 2 DM [49], and high glucose concentrations altered the content and distributions of three tubulin isotypes, reducing RBC deformability and osmotic resistance [50]. Moreover, the RBC abnormalities in the membrane lipid composition, associated with some perturbations, can significantly affect RBC receptor binding and enzyme activities, as it occurs in the diabetic retinopathy, where higher amounts of phosphatidyl-choline species have been found in RBC [51].

In conclusion, this study provides the first documented findings giving strong evidence of a putative role of RBC as an extra-hepatic mechanism for controlling serum levels of nitrogen-related metabolites, which differs according to gender in healthy subjects. Type 2 DM promotes a characteristic pattern of metabolic disturbances that culminates in a loss of the differential management of nitrogen-related metabolites seen in healthy women and men. Therefore, it is not unlikely that these characteristic patterns of blood metabolites elicited by type 2 DM might be involved in the specific physiopathology of this disease, and changes in the oxidative status, i.e., increased lipid peroxidation in RBC membranes of diabetic patients, are playing a role in the metabolic alterations found in these blood cells.

## Supporting information

S1 File Descriptive statistics. (XLS)

S2 File Best fit distributions for all determinations. The table reports D-statistics and p-values of the KS goodness of fit test for each of the 84 data sets. The KS test for goodness of fit performs a test of the distribution of an observed random variable against a given distribution under the null hypothesis that the two distributions are identical. The table also reports the MLEs for shape, location and scale parameters obtained from the maximum likelihood estimation needed to perform the KS tests. We fitted the data to the following distributions: beta, exponential, exponential-Weibull, exponential-power law, Gilbrat, logistic, lognormal, normal, Pareto, power law, Weibull minimum, and Weibull maximum.
(PDF)

S3 File Matrix containing the Spearman Rho and P values. (XLS)

S4 File Table 1 with “normal” statistics. Results are expressed as means ± SD. We used the Student’s unpaired t-test and the Mann-Whitney test to compare a continous variable between the two groups. Thereafter, these differences were contrasted with t-test for paired data.
(TIF)
