# Supplementary material for: The role of oxidant stress and gender in the erythrocyte arginine metabolism and ammonia management in patients with type 2 diabetes
Source: PLoS One. 2019 Jul 17;14(7):e0219481. doi: 10.1371/journal.pone.0219481 (PMC6636741; doi:10.1371/journal.pone.0219481)
Supplement: S2 File — The table reports D-statistics and p-values of the KS goodness of fit test for each of the 84 data sets. The KS test for goodness of fit performs a test of the distribution of an observed random variable against a given distribution under the null hypothesis that the two distributions are identical. The table also reports the MLEs for shape, location and scale parameters obtained from the maximum likelihood estimation needed to perform the KS tests. We fitted the data to the following distributions: beta, exponential, exponential-Weibull, exponential-power law, Gilbrat, logistic, lognormal, normal, Pareto, power law, Weibull minimum, and Weibull maximum. (PDF) [file pone.0219481.s002.pdf]

Supplement 2.

Best fit distributions for all determinations.
The table reports D-statistics and p-values of the KS goodness of fit test for each of the 84 data sets. The KS test for goodness of fit performs a test of the distribution of an observed random variable against a given distribution under the null hypothesis that the two distributions are identical. The table also reports the MLEs for shape, location and scale parameters obtained from the maximum likelihood estimation needed to perform the KS tests. We fitted the data to the following distributions: beta, exponential, exponential-Weibull, exponential-power law, Gilbrat, logistic, lognormal, normal, Pareto, power law, Weibull minimum, and Weibull maximum.

M: MALE
F:FEMALE

Goodness of fit test results									
			**Best fit distribution**	**KS test**		**Best fits MLEs (shape, c, location, and scale)**			
		**Gender**		**D-statistic**	**p-value**	**S**	**C**	**LOC**	**SCALE**
**Arginine**									
Control	RBC	M	lognormal	0.16428	0.20659	0.376438		1.308643	5.47568
		F	lognormal	0.1665159	0.194354	1.29766		3.436246	0.67795
	Serum	M	logistic	0.1058	0.76317			0.18254	0.037755
		F	Power law	0.149034	0.306546	1.45524		0.0614427	0.207557
Diabetics	RBC	M	logistic	0.123167	0.548778			5.46838	0.747606
		F	logistic	0.185849	0.110702			7.314805	1.144642
	Serum	M	lognormal	0.131201	0.4633701	0.501445		0.01789	0.114442
		F	Exponential weibull	0.1779749	0.1402727	264.953	0.348029	0.056437	0.00075

**Nitrites**									
Control	RBC	M	logistic	0.172549	0.164134			117.5069	23.61217
		F	Exponential power law	0.17374357	0.15862432	0.606062		71.99	61.42386
	Serum	M	Gilbrat	0.16669	0.19339			4.0619	11.44269
		F	Exponential weibull	0.244136	0.01386	188.6350	0.7192228	1.756299	1.16123
Diabetics	RBC	M	Normal	0.13516759	0.424589			63.72999	14.67089
		F	logistic	0.140824	0.373169			72.97857	16.18652
	Serum	M	Exponential weibull	0.1201168	0.58358	7.625178	0.7439537	2.57963	13.2755
		F	logistic	0.21327	0.0447518			53.33497	10.4515
**Citrulline**									
Control	RBC	M	normal	0.105069	0.77309			141.40	47.50778
		F	logistic	0.2543139	0.009089			181.1275	9.6051
	Serum	M	logistic	0.13439	0.4319			243.0979	21.0271
		F	logistic	0.20107	0.068019			225.874	33.9907
Diabetics	RBC	M	logistic	0.14268	0.357215			294.8807	40.30802
		F	logisic	0.3039	0.00089			350.9909	43.272149
	Serum	M	logistic	0.1356	0.420248			569.447	138.6046
		F	logistic	0.19474	0.08367			663.5169	126.9296
**Ornithine**									
Control	RBC	M	normal	0.15339	0.2749			181.375	33.34268
		F	logistic	0.2162146	0.04031			121.2159	22.90108
	Serum	M	beta	0.13549	0.421465	0.779	0.80878	40.999	53.0000
		F	logistic	0.2164117	0.040027			58.57125	5.34658

Diabetics	RBC	M	beta	0.1592246	0.2364787	0.926456	0.876421	111.78034	147.219
		F	beta	0.180789	0.129046	5.033445	5.316408	37.070529	361.411
	Serum	M	beta	0.11707	0.619573	33.18864	118.13943	-14.5890	396.721
		F	lognormal	0.10228639	0.7967729	0.155339		-50.3064	110.7159
**Ammonia**									
Control	RBC	M	normal	0.11059	0.70025			283.69	96.0086
		F	Weibull min	0.1875639	0.1049	8.09089		-448.231	817.935
	Serum	M	logistic	0.11757	0.61361			50.217	11.7051
		F	logistic	0.13425178	0.4333437			47.37623	11.17336
Diabetics	RBC	M	exponential	0.1260418	0.51717			421.9	110.3324
		F	beta	0.20026	0.069869	0.546337	0.7173	151.3328	635.667
	Serum	M	Exponential weibull	0.115537	0.6382	172.4512	0.31065	17.6645	0.25214
		F	Exponential weibull	0.16989	0.1769	0.7432	4.3299	26.99329	133.655
**Glucose**									
Control	Serum	M	logistic	0.081408944596202959	0.95362559191967011			93.725473513127511	6.2381507297077894
		F	beta	0.063350994697624063	0.99712418497442634	1.3749395301794118	1.6069798014712964	72.619931937412161	33.226373503722144
Diabetics	Serum	M	logistic	0.090438340854283983	0.89906823249140322			138.91353881897874	13.274829228028919

		F	logistic	0.090438729689889474	0.89906539365009153			145.9135168365886	13.274828567766228
**Triglycerides**									
Control	Serum	M	beta	0.066488671353012174	0.99443661380517578	1.3080556507522143	2.228276396322534	117.46346521215393	88.21505301537151
		F	lognormal	0.050725721131268608	0.99995134248772533	0.28008562832108197		82.668758514730399	58.269274000802035
Diabetics	Serum	M	Power law	0.072821242806068831	0.98378594431699085	1.0503315544364957		110.83600096980945	117.16399904116679
		F	logistic	0.12537822785665176	0.5243675007596269			180.3241208668489	18.771454092377226
**AST**									
Control	Serum	M	logistic	0.08826	0.91433			16.88085	2.46169
		F	Exponential weibull	0.1074	0.741336	62.25524	0.371341	16.064	0.14432
Diabetics	Serum	M	lognorm	0.08239	0.9488389	0.048275		-115.279	135.1256
		F	logistic	0.09083	0.896173			22.86877	5.9587

**ALT**									
Control	Serum	M	beta	0.0911538	0.8937	0.82809	0.834268	10.82046	23.1795
		F	lognormal	0.108019	0.733746	0.53737		4.58654	18.59916
Diabetics	Serum	M	lognormal	0.078179	0.96738	0.4835		3.714734	13.6584
		F	exponencial	0.0798559	0.96062			13.99999	11.00000
**Urea**									
Control	RBC	M	logistic	0.055941	0.999628			1.090576	0.138671
		F	Weibull max	0.0831389	0.944997	4.696647		2.359825	1.3690001
	Serum	M	Exponential weibull	0.1365117	0.411958	241.607	0.31885	0.09082	0.00098
		F	Pareto	0.125705	0.520812	1.722121		-0.07716	0.30716
Diabetics	RBC	M	Exponential power law	0.0893481	0.9068708	0.801215		1.429999	1.1357832
		F	Power law	0.120292	0.5815448	0.513628		1.399999	3.986721
	Serum	M	Weibull max	0.1346946	0.429095	1.273849		2.853879	1.220335
		F	Lognormal	0.178396	0.1385406	1.777346		0.20823	0.178658

**Uric Acid**									
Control	RBC	M	Beta	0.0727379	0.98398	1.180507	1.204808	89.513640	91.0476
		F	Power law	0.0740678	0.980643	1.027318		75.86255	77.137443
	Serum	M	Normal	0.052033	0.999914			179.0	28.64698
		F	Beta	0.0531758	0.99986	1.47233	1.472038	93.82706	122.342
Diabetics	RBC	M	Power Law	0.065469	0.99546	1.054462		106.8278	112.17212
		F	Logistic	0.0780	0.9680579			124.7667	14.00563
	Serum	M	Normal	0.096925	0.846604			115.0	19.77498
		F	Logistic	0.05525487	0.9997059			99.08868	7.923599
**Insulin**									
Control	Serum	M	Gilbrat	0.1052792981056267	0.77027253797919037			0.8046671667729976	3.96427034267
		F	Exponential weibull	0.10652752873175297	0.75352676074713054	0.549119269432	1.6081461296582	0.89999999999	11.1715086177
Diabetics	Serum	M	Exponential power law	0.10763362428626033	0.73883713800173378	0.86535792266914457		0.8999999999999999	15.9906963737317 3
		F	Exponential power law	0.11440802219086182	0.65209913362864591	0.90456620109096519		0.59999999999999987	15.937329905210767

**Cholesterol**									
Control	Serum	M	Logistic	0.09065897243857	0.89745109483425			138.249310229853	7.87133112541001
		F	Logistic	0.07312066557571	0.98306621579965			148.643949735496	9.11033657567190
Diabetics	Serum	M	Logistic	0.09502613675456	0.86295847141137			175.273625810287	13.7527295581938
		F	Beta	0.0677095554162	0.99298997054454	1.94858874571129	1.56851516583062	143.590289590161	74.9250143789352
**Arginase**									
Control	RBC	M	Beta	0.10449870744279033	0.78083346450110724	2.03025169156778 37	1.87445795623585 52	1.50779831894966 82	2.1297500482998735
		F	Exponential power law	0.06545315804463 4585	0.99547597058805426		1.33371176387156 08	0.869440755586701 97	2.0096885672227129
Diabetics	RBC	M	Esponential weibull	0.08801536214348 6819	0.91597406105375323	0.16180600784294674	6.12818021597617 25	1.25892672047072 55	9.2735610116041869
		F	Exponential Weibull	0.09753942888173 1505	0.84115493599831437	42.69124775675327	0.501408531388265 24	1.26521150194846 93	0.12664545234472205
MLEs: Maximum likelihood estimators for shape, location and scale parameters for data of each determination according to gender.									
