# Supplementary figures and images for: The role of oxidant stress and gender in the erythrocyte arginine metabolism and ammonia management in patients with type 2 diabetes
Source: PLoS One. 2019 Jul 17;14(7):e0219481. doi: 10.1371/journal.pone.0219481 (PMC6636741; doi:10.1371/journal.pone.0219481)

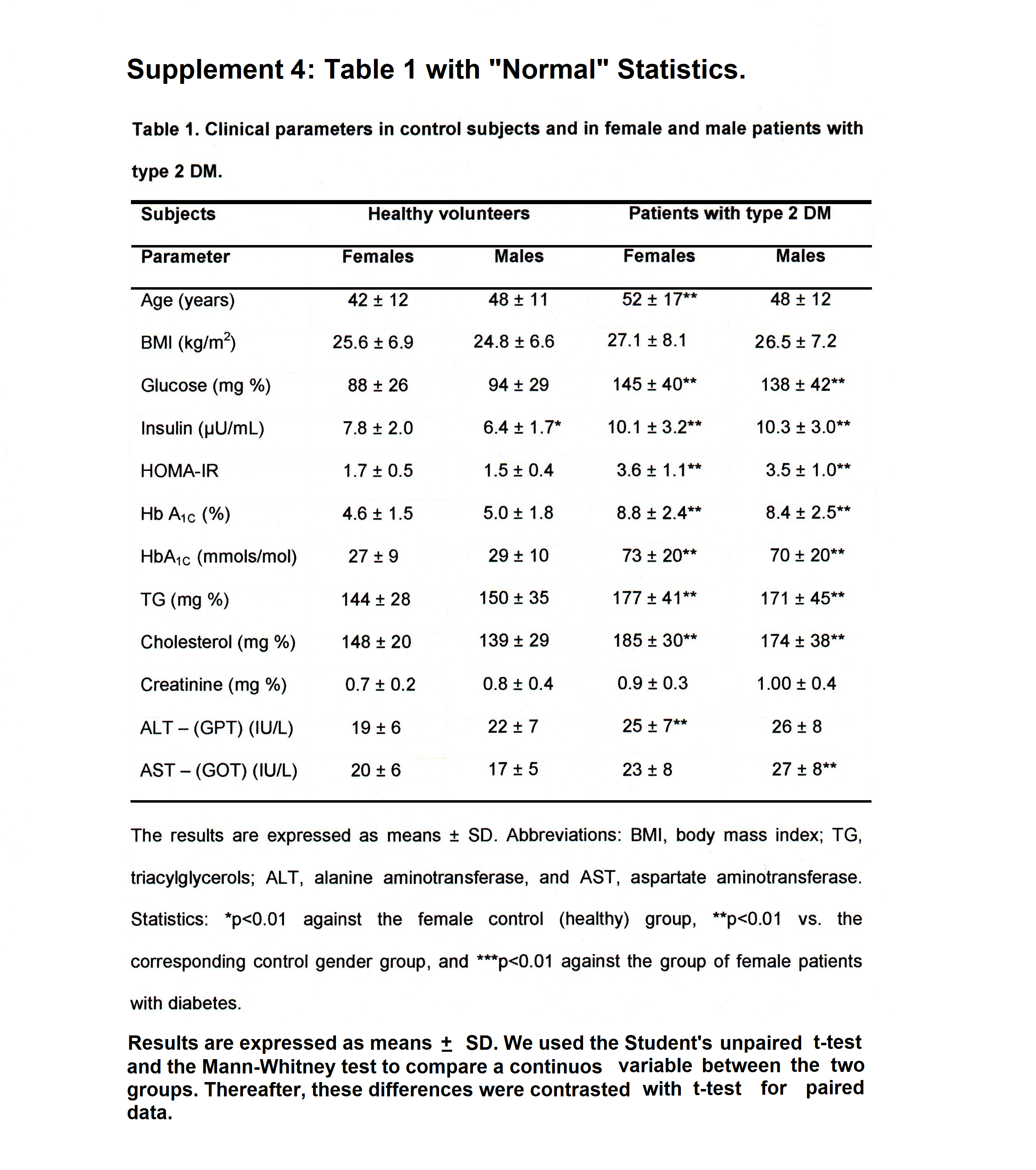

Supplement: S4 File — Results are expressed as means ± SD. We used the Student’s unpaired t-test and the Mann-Whitney test to compare a continous variable between the two groups. Thereafter, these differences were contrasted with t-test for paired data. (TIF) [file pone.0219481.s004.tif]
